# Promoter methylation and expression of TIMP3 gene in gastric cancer

**DOI:** 10.1186/1746-1596-8-110

**Published:** 2013-07-02

**Authors:** ZhiYu Guan, Jun Zhang, ShiHui Song, DongQiu Dai

**Affiliations:** 1 Department of Cardiovascular and Thoracic Surgery, TianJin Medical University General Hospital, 154 AnShan Road, HePing Section, TianJin 300052, China; 2 Department of Oncology, the First Affiliated Hospital of China Medical University, 155 Nanjing Beijie, Heping Section, Shenyang, Liaoning Province 110001, China

**Keywords:** Gastric Adenocarcinoma, Tissue Inhibitor of Metalloproteinase 3 (TIMP3), Methylation

## Abstract

**Background:**

Gastric carcinoma development is a multi-stage process that involves more than one gene. Aberrant changes in DNA methylation are considered as the third mechanism that leads to anti-oncogene inactivation, which plays an essential role in tumor development. In this study, we assessed the relationship among the aberrant methylation of the promoter CpG islands of tissue inhibitor of metalloproteinase 3 (TIMP3) gene, its protein expression, and the clinicopathological features of gastric adenocarcinoma.

**Methods:**

The methylation status of the promoter CpG islands and the protein expression of TIMP3 gene in tumors and adjacent normal mucosal tissues of 78 patients with gastric adenocarcinoma were detected by methylation-specific PCR (MSP) and immunohistochemistry.

**Results:**

The CpG island methylation of TIMP3 was detected in tumor tissues, cancer-adjacent tissues, and lymph nodes with metastasis. In increasing order, the hypermethylation frequency of these tissues were 35.9% (28 of 78 non-neoplastic tissues), 85% (17 of 20 early-stage cases), 89.7% (52 of 58 progressive-stage cases), and 100% (78 of 78 metastatic lymph node). A marked difference was found between tumors and non-neoplastic tissues (*P* < 0.05), but no difference existed among the subgroups of tumors (*P* > 0.05). Immunohistochemistry analysis confirmed TIMP3 down-regulation in tumor tissues. The rate of TIMP3 gene expression was 100% in non-neoplastic tissues but apparently decreased to various extents at different stages, i.e., decreased to 30% (6/20) at the early stage, to 3.4% (2/58) at the progressive stage, and to 0% (0/78) in metastatic lymph nodes. Among the 70 tumor tissues with negative TIMP3 expression, 64 (91.4%) were hypermethylated and 6 were unmethylated (8.6%), indicating a significant association between hypermethylation and reduced or negative TIMP3 expression (*P* < 0.01).

**Conclusion:**

The hypermethylation of the promoter region in CpG islands is the main mechanism of TIMP3 gene expression and may provide evidence for the molecular diagnosis and stage evaluation of gastric cancer.

**Virtual slides:**

The virtual slides for this article can be found here: http://www.diagnosticpathology.diagnomx.eu/vs/1756134016954958

## Background

Gastric cancer (or gastric carcinoma) is one of the most common malignancies in the world. Disease development in gastric cancer is a multi-stage process that involves more than one gene. Disease factors ultimately act on different genes at different stages, causing a change in gene structure and expression levels that lead to the development of gastric carcinogenesis. Loss of function of an anti-oncogene may occur through various channels. In addition to deletions and mutations, aberrant changes in DNA methylation are considered as the third mechanism leading to anti-oncogene inactivation [1,2], which plays an essential role in tumor development. CpG islands, which are CpG-rich sequences located in the upstream promoter region of a housekeeping gene, are regions at which cytosine methylation does not generally occur, although stimulation by certain factors leads to its methylation. Consequently, DNA expression is inhibited in this region [3]. The inactivation of many tumor-associated genes such as P16, THBS1, TIMP3, hMLH1, and MGMT are related to the methylation of their respective promoter regions [4-8]. Tissue inhibitor of metalloproteinase-3 (TIMP3) is related to tumor development, particularly antagonizing the activity of matrix metalloproteinases as well as inhibiting tumor growth, angiogenesis, invasion, and metastasis [9].

In this work, we detected the methylation of the TIMP3 gene promoter CpG island and protein expression in normal gastric mucosa tissues, gastric cancer tissues, and metastatic lymph nodes of 78 patients with gastric cancer using methylation-specific PCR (MSP) and immunohistochemistry. We explored the correlation of gene promoter methylation with its corresponding protein expression and then analyzed the relationship of TIMP3 gene CpG island abnormal methylation with the development, as well as clinical and pathological features, of gastric adenocarcinoma.

## Materials and methods

### Materials

All 78 cases of gastric normal tissue, gastric carcinoma, and metastatic lymph nodes were verified by pathological diagnosis. The tumor samples were obtained between March 1998 to May 2005 at the First Affiliated Hospital and Liaoning Provincial Tumor Hospital from postoperative patients with gastric cancer (male: *n* = 54; female: *n* = 24) with a mean age of 56.2 ± 7.5 years. Among the samples obtained, 20 were of early gastric cancer, 58 were of advanced gastric cancer, 44 were of differentiation, and 14 were undifferentiated. Tumor staging was based on the International Union Against Cancer TNM staging standards, Typing of Gastric Cancer Growth Way and Statute of Staging, and gastric cancer typing in Japan [10-12] from the China Medical University Cancer Institute. Hydroquinone (10 mmol/L) and sodium bisulfate (3.9 mol/L) were purchased from Sigma. A DNA Clean-up System was purchased from Promega. TIMP3 antibody and SP kit were purchased from Boster Biological Engineering Co., Ltd. (Dalian). All specimens were handled and made anonymous according to the ethical and legal standards. The protocol was approved by the Medical Ethics Committee of the Liaoning Provincial Tumor Hospital and China Medical University.

### MSP

In the MSP method [13], methylase processing was conducted on normal peripheral blood cells and used as positive controls. Untreated cells were used as negative controls. The sequences of the methylated (TIMP3-M) and unmethylated (TIMP3-U) primers were as follows: (1) TIMP3-M primer sequences, 5′CGTTTCGTTATTTTTTGTTTTTCGGTTTTC-3′ and 5′-CCGAAAACCCCGCCTCG-3′; and (2) TIMP3-U primer sequences, 5′-TTTTGTTTTGTTATTTTTTGTTTTTGGTTTT-3′ and 5′-CCCCCCAAAAACCCCACCTCA-3′. The PCR reaction conditions were as follows: 95°C initial denaturation for 5 min, 95°C, denaturation for 30 s (40 cycles), 59°C annealing for 60s (40 cycles), 72°C extension for 60s (40 cycles), and 72°C extension for 10 min. Agarose gel electrophoresis and EB staining were then performed. Data from the gels were collected with a laser density scanner (Pharmacia LKB Ultroscan) and subsequently analyzed. All experiments were repeated three times, and the mean value was used for statistical analysis.

### Immunohistochemistry

For immunohistochemistry, we used the streptomycesavidin–peroxidase staining method. Immunohistochemical staining was performed according to the manufacturer’s instructions. As a diagnostic criterion, the observation of a brownish yellow cytoplasm after staining was considered a positive result for TIMP3 expression. For the controls, a normal tissue area was stained as the positive control, and a normal tissue section was used as the negative control and stained with PBS instead of a primary antibody. Scoring standard was performed according to the method described by Shimizu [14]. We observed the staining intensity and distribution of positive cells at high magnification; no staining was scored as 0, weak staining but significantly stronger than the negative control was scored as 1, a clear stain is scored as 2, and a strong stain is scored as 3. No positive control cell is scored as 0. The required number of positive cells, i.e., <30%, 30%–60%, and >60%, were scored as 1, 2, and 3, respectively. Based on the total scores, the samples were assessed for TIMP3 expression. A score of ≤2 was classified as negative, a score of 3 was weakly positive, a score ranging from 4 to 5 was positive, and a score of 6 was strongly positive.

### Statistical analysis

The differences among the TIMP3 gene promoter methylation rate and protein expression of the samples were detected and statistically analyzed by the *χ*^2^ and Fisher methods. All data were analyzed with SPSS12 statistical software.

## Results

### TIMP3 gene promoter methylation analysis

**Figure 1:**
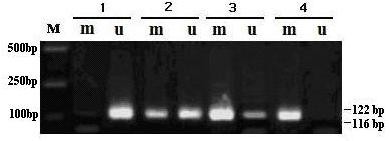
**Methylation-specific PCR (MSP): 1, normal control (normal gastric tissue); 2, early gastric cancer; 3, advanced gastric cancer; 4, transfer of lymph node. M**, Marker DL2000; **m**, Methylation amplification fragment; **u**, unmethylation amplification fragment.

Extracted genomic DNA from the sample was amplified by MSP. The sizes of the methylated and unmethylated PCR products were 116 and 122 bp, respectively. TIMP3 gene promoter methylation phenomenon was generally observed in normal gastric tissues, gastric carcinoma, and lymph node metastasis samples. The positive detection rates were as follows: 85% (17/20) for early gastric cancer, 89.7% (52/58) for advanced gastric cancer, 98.7% (77/78) for metastatic lymph nodes, and only 35.9% (28/78) for the normal gastric tissue. A significant increase in TIMP3 gene promoter methylation was observed in all gastric cancer groups (*P* < 0.01; Figure 1 and Table 1), suggesting that the methylation of TIMP3 gene may be involved in the carcinogenesis and metastasis of gastric cancer tumors and metastatic lymph node tissues.

**Table 1:** Gastric carcinoma TIMP3 promoter methylation and protein expression

**Organizations**	***n *(%)**	**TIMP3 promoter methylation**	**TIMP3 protein expression**
		**Positive**	**Positive**
Normal stomach	78	28(35.9)	78(100.0)
Early gastric cancer	20	17(85.0)	9(45.0)
Advanced gastric cancer	58	52(89.7)	21(36.2)
Lymph node metastases	78	77(98.7)	12(15.4)

### Immunohistochemistry analysis of TIMP3 protein expression

**Figure 2:**
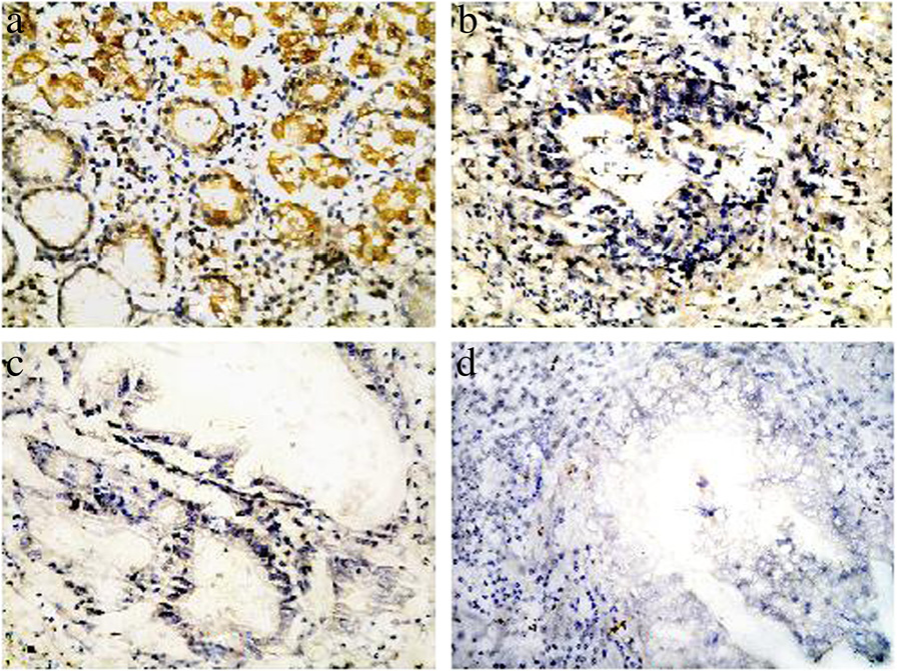
TIMP3 protein immunohistochemisty (SP 400×): a, normal gastric tissue; b, early gastric cancer; c, advanced gastric cancer; d, transfer of lymph node.

TIMP3 protein expression was positive in all 78 cases of normal gastric tissue (100%). Among the gastric carcinoma cases, 9 in 20 patients were positive for early gastric cancer; 11 cases were negative (55%), 21 cases were positive, and 37 were negative (63.8%) in 58 cases of advanced gastric cancer; and 12 cases were positive and 66 cases were negative (84.6%) in 78 patients with metastatic gastric lymph nodes. The reduced TIMP3 expression that was observed in the gastric cancer groups was found to be statistically significant (*P* < 0.01; Figure 2 and Table 1), suggesting that TIMP3 protein expression rate decreased in gastric cancer tumors and metastatic lymph node tissues. This reduced expression of the gene protein may be due to the previously observed increase in TIMP3 methylation in the gastric cancer samples because methylation is known to suppress protein expression. TIMP3 protein expression was mainly located in the cytoplasm of normal glands. In a few cells, a small amount of expression was found in the cell membranes (Figure 2). In the normal gastric mucosal cells, no glandular structure destruction was observed and coloring was not significantly reduced. The early gastric cancer glands showed structural damage and weak cancer cell coloring. The advanced gastric cancer tissues showed large areas of massive growth (undifferentiated carcinoma) and, apart from the individual cells, the rest were not colored.

### Relationship between TIMP3 gene promoter methylation and TIMP3 protein expression

In the advanced gastric cancer group, the rate of TIMP3 methylation was significantly higher than the lumpish and nested growing tissues (100% vs. 77.8%, respectively; *P* < 0.01). The TIMP3 methylation rate in metastatic lymph nodes in the ≥7 tissue group was significantly higher than the <7 tissue group (100% vs. 83.3%, respectively; *P* < 0.05). The subserosal and serosal tissue group was significantly higher than the submucosal and myometrium tissue group (91.3% and 96.6% vs. 50%, respectively; *P* < 0.05). Increased TIMP3 gene methylation rate was generally observed in diffuse-like growth, lymph node metastases (≥7), and subserosal and serosal tumors, implying that no relationship existed between TIMP3 gene methylation and tumor size, macroscopic type, and histological type. The positive rate of the 58 cases of advanced gastric cancer tissues was 36.2% (21). TIMP3 protein expression in massive and nested growing tissues was significantly higher than that of diffuse-like growth tissues (55.6% vs.19.4%, respectively; *P* < 0.01). TIMP3 protein expression in the metastatic lymph node ≥7 tissues was significantly higher than the <7 tissue (47.2% vs. 18.2%, respectively; *P* < 0.05) (Table 2). These results suggested that decreased TIMP3 protein expression rate was more common in diffuse-like growth and lymph node metastatic (≥7) tumors, but reduced expression was not related to tumor size, gross type, histological type, and depth of invasion.

**Table 2:** Relationship between advanced gastric cancer pathology TIMP3 methylation and its protein expression level

**Index**		***n***	**TIMP3 promoter methylation**	**TIMP3 protein tumor size**
Tumor size (cm)	<5	5	3 (60.0)	1 (20.0)
	5 − 10	45	42 (93.3)	18 (40.0)
	>10	8	7 (87.5)	2 (25.0)
General types	Borr.2	12	10 (83.3)	4 (33.3)
	Borr.3	37	33 (89.2)	15 (40.5)
	Borr.4	9	9 (100.0)	2 (22.2)
Histological differentiation	Differentiated	44	39 (88.6)	17 (38.6)
	Undifferentiated	14	13 (92.9)	4 (28.6)
Growth pattern	Clumps + nested	27	21 (77.8)	15 (55.6)
	Diffuse-like	31	31 (100.0) ^b^	6 (19.4) ^b^
Lymph node metastasis	<7	36	30 (83.3)	17 (47.2)
	≥7	22	22 (100.0) ^a^	4 (18.2) ^a^
Infiltration	Submucosal muscularis	6	3 (50.0)	1 (16.7)
	Subserosal	23	21 (91.3) ^a^	9 (39.1)
	Serosa	29	28 (96.6)	11 (37.9)

^a^*P* < 0.05, ^b^*P* < 0.01.

## Discussion

The formation of malignancy is multi-factorial and occurs in multiple stages. The biological characteristics of malignant tumors include invasive growth and metastasis. The mechanism behind tumor development involves abnormal changes in gene structure and function in the interior of the cell. Modern theorists consider that the process of tumor formation comprises two major mechanisms: the first one is at the genetic level, i.e., gene mutation causes DNA nucleotide sequence changes; and the second one is at the epigenetic level. Previous studies have focused on changes in gene expression that are inherited through meiosis and do not involve a change in DNA sequence but affect the expression and gene regulating function of DNA, mainly by chemical modification. This mechanism is gaining increased attention from researchers of tumor formation processes [15,16].

In the DNA of tumor tissues, abberant methylations are often observed in gene promoter regions, including hypomethylation in the oncogene. This gene is closely related to cell proliferation cycle and hypermethylation in tumor suppressor genes. These abnormal methylation changes can activate oncogenes [17-19] and inhibit the mRNA transcription of tumor suppressor genes [20], leading to gene inactivation. Aberrant methylation in DNA occurs through the presence of highly methylated CpG islands in the 5′ end of a promoter control region. The TIMP family has four protein members: TIMP-1, 2, 3, and 4. TIMP-1, 2, and 4 are soluble secretory proteins. By contrast, TIMP-3 is a non-soluble protein that combines with the extracellular matrix, is located in the outer cell membrane [21], and can be closely connected with the basilar membrane. The function of TIMPs is the inhibition of tumor necrosis factor-α (TNF-α)-converting enzymes and the induction of programmed cell death through the stable cell surface TNF-α receptor [22]. The induction of apoptosis through TIMP occurs by two pathways: MMP dependent and independent. Although TIMP-3 and 4 have a high affinity for MMP-2, the proteins effectively inhibit MT1-MMP, thereby activating the MMP-2 zymogen process and inhibiting tumor cell growth and metastasis. Smith et al. [23] found that the expression of TIMP-3 in human colon cancer cell lines are rare in mitosis. Most cells are arrested in the G1 phase, although the application of TNF-α antibody decreases mitotic arrest by 70%.

To explore the relationship between TIMP3 and gastric cancer, as well as its mechanism in gastric inactivation, we detected TIMP3 gene 5′ CpG island hypermethylation in normal gastric mucosa, gastric carcinoma, and metastatic lymph nodes. We found that in >85% of all gastric cancer tissues, the TIMP3 gene 5′ CpG island exhibited aberrant methylation that was significantly higher (*P* < 0.01) than that of the normal gastric tissue group, which implied that TIMP3 gene promoter CpG island methylation reduced TIMP3 gene expression and was thus the main tumor suppressor-inactivation mechanism in gastric cancer. Gagnon et al. [24] reported that in all MCF-7 lung cancer cell lines with TIMP3 mRNA expression loss and TIMP3 gene promoter CpG island methylation, demethylation was induced by 5-AZA-CdR. TIMP3 expression before and after 5-AZA-CdR induction was assessed by RT-PCR, which showed that demethylation resulted in TIMP3 gene re-expression, consistent with our results.

In the present study, all 78 cases of normal gastric tissue were positive for TIMP3 protein expression. Among these 78 cases, only 28 (35.9%) were positive for methylation. Among the 20 patients with early gastric cancer, 9 were positive for TIMP3 protein expression and 17 (85%) were positive for methylation. Among the 58 cases of advanced gastric cancer, 21 were positive for TIMP3 protein expression and 52 (89.7%) were positive for methylation. Among the 78 cases of metastatic lymph nodes, 12 were positive for TIMP3 protein expression and 77 cases (98.7%) were positive for methylation. Increased methylation rate was significant among the early cancer, advanced cancer, and metastatic lymph node samples compared with normal tissue samples (*P* < 0.01), confirming that the loss of TIMP3 protein expression was closely related to TIMP3 gene promoter CpG island methylation. Feng HF et al. [25] reported the same results in JAR and JEG-3 choriocarcinoma cell lines. Therefore, our study confirmed that TIMP3 gene promoter CpG island methylation was the main reason for the reduced TIMP3 protein expression in gastric cancer.

Among the 58 advanced gastric cancer samples, 52 were positive for TIMP3 promoter CpG island methylation and 21 were positive for TIMP3 protein expression. This finding suggested that apart from CpG island methylation, other factors such as genetic variation caused the absence of TIMP3 gene expression. In our experiments, 34 samples were positive for methylation, and unmethylation was usually interpreted as follows: incomplete methylation status of genes may exist, and if the tumor tissue is mixed with non-tumor cells, a positive PCR product for unmethylation (by MSP with unmethylated primer) may be observed. TIMP3 protein expression of the sample was negative, so these samples were defined as methylation positive.

In China, the 5-year survival rate of gastric carcinoma patients is only about 40% after resection. The poor prognosis is associated with extensive local invasion and/or regional lymph node metastasis [26]. Local recurrence remains as a major cause of cancer-related deaths after resection in gastric cancer patients [27]. Therefore, reliable criteria for the prediction of recurrence and identification of tumors must be established to understand molecular and cellular processes, as well as discover new and possible therapeutic molecular targets [28].

Kerbel [29] reported that subcloning tumor cells with metastatic potential have a growth advantage in the early stage of primary foci. The reason for such growth advantage is that the metastatic subclone cell population may secrete a particular factor or local cell growth factor, thereby causing a special reaction that results in selective growth. Detection of molecular marker levels in primary tumor specimens may reflect the general metastatic characteristics of the whole tumor. In this group, the methylation rates were only 35.9% for the normal gastric tissues, 85% and 89.7% for the early and advanced cancers, respectively, 98.7% for metastatic lymph nodes. Consistently high TIMP3 protein expression was observed only in normal gastric tissues. Early and advanced gastric cancers exhibited focal weak expression, and metastatic lymph nodes showed almost no positive expression. This finding suggested that TIMP3 methylation increased with tumor progression and that protein expression gradually decreased. Consequently, the metastatic potential and growth advantage of cells gradually increased, ultimately resulting in metastasis. Yegnasubramanian et al. [30] reported that tumor metastasis was related to TIMP3 methylation in prostate cancer cell lines as revealed by the real-time MSP of samples from 73 patients with early prostate cancer, 91 patients with metastatic prostate cancer, and 25 cases with prostatic tissue. This result implied a relationship between CpG methylation and the grading and progress of prostate cancer. TIMP3 gene methylation reportedly plays an important role in the metastatic process, and the detection of TIMP3 CpG methylation has some practical value in predicting the metastatic potential of the primary tumor.

TIMP3 has a high frequency of genetic variation in many types of human malignant tumors, including mutations, deletions, methylation, chromosomal translocation, and so on [31]. These mutations result in the loss of the normal function of a tumor suppressor gene, namely, the inhibition of cell proliferation (i.e., gene inactivation) and are closely related to the development of cancer [32]. However, few studies have been conducted on TIMP3 gene modification in primary gastric cancer, and the results of these studies show discrepant results from a low gene deletion rate to no mutation at all. Accordingly, other inactivation mechanisms of gastric cancer involving TIMP3 gene have been investigated [33]. Thus, the complexity of tumor pathogenesis is not only a reflection of genetic change by mutation or deletion but also a reflection of epigenetic alterations, such as DNA methylation. An in-depth study of the relationship between DNA methylation and gene expression, as well as its corresponding mechanism, is recommended because the results can guide the clinical treatment of cancer.

## Competing interest

The authors hereby declare that they have no competing interests with one another.

## Authors’ contributions

ZG and JZ carried out the pathological and PCR examinations, DD and ZG analyzed the data, and ZG drafted the manuscript. All authors read and approved the final manuscript.
